# Partial Resection of the Kidney

**Published:** 1919-01

**Authors:** 


***Partial Resection of the Kidney***. By Dr. SIMONIN. *Bulletin de la Société de Chirurgie de Paris*, May 21, 1918.

Dr. Simonin relates the case of a soldier presenting exceptionally numerous wounds :

1) A penetrating wound in the left lumbar region. X-rays detected a projectile lodged below the diaphragm; there was blood in the urine.

2) A penetrating wound in the posterior part of the perineal region in which the projectile was detected by X-rays.

And six other severe penetrating wounds in both legs and in the right arm, together with a reddish edema of both feet.

Most of these injuries necessitated surgical intervention.

The first operation was a partial resection of the kidney, injured by the lumbar projectile. A shell-fragment as big as one half of the thumb was discovered in the upper part of the kidney. The fragment was removed, and all bruised tissues were cut off. A suture of the kidney wound was performed with catgut. No hemorrhage occurred after this intervention, and the wound healed quite satisfactorily. But of course this soldier had so many other injuries that his state did not improve simply. The left leg had to be amputated. But after five months the lumbar fistula was shut, and that wound completely healed; the general condition was excellent.

